# Can Vitamin D Supplementation Improve Inflammation in Relapsing-Remitting Multiple Sclerosis Patients?

**DOI:** 10.3390/biomedicines12071580

**Published:** 2024-07-17

**Authors:** Martyna Lis, Natalia Niedziela, Jowita Adamczyk-Zostawa, Krzysztof Wierzbicki, Zenon Czuba, Jolanta Zalejska-Fiolka, Wojciech Bartman, Agata Świętek, Monika Adamczyk-Sowa

**Affiliations:** 1 Department of Neurology, Faculty of Medical Sciences in Zabrze, Medical University of Silesia, 40-055 Katowice, Polandm.adamczyk.sowa@gmail.com (M.A.-S.); 2 Department of Ophthalmology, Faculty of Medical Sciences in Zabrze, Medical University of Silesia, 40-055 Katowice, Poland; 3 Department of Microbiology and Immunology, Faculty of Medical Sciences in Zabrze, Medical University of Silesia, 40-055 Katowice, Poland; 4 Department of Biochemistry, Faculty of Medical Sciences in Zabrze, Medical University of Silesia, 40-055 Katowice, Poland; 5 Silesia LabMed Research and Implementation Center, Medical University of Silesia in Katowice, 19 Jordana St., 41-808 Zabrze, Poland; agata.swietek@sum.edu.pl

**Keywords:** multiple sclerosis, inflammation, immune system, vitamin D, supplementation

## Abstract

(1) Background: Studies indicate that vitamin D (VitD) may reduce inflammation in multiple sclerosis (MS). The aim of the study was to assess the effect of supplementation with different doses of VitD on inflammation in relapsing-remitting MS (RRMS) patients. (2) Methods: The effect of 6-month supplementation with different doses of oral VitD (2000 IU/day) in a high-dose group (HD, *n* = 23) and a low-dose group (15,960 IU/month) (LD, *n* = 29) on selected markers of inflammation was assessed in 52 RRMS patients. (3) Results: Females constituted the majority of participants (63.46%). The median age [years] was 39.5 [34.5–49.8] and 47 [40.0–55.0] in the HD and LD groups, respectively. Significant differences were observed in age (*p* = 0.028), body weight (*p* = 0.014) and height (*p* = 0.001) between the study groups. Considering the BMI, statistically significant differences were not found (*p* = 0.496). The median 25(OH)D concentration [ng/mL] increased from 23.023 [15.578–25.76] in the HD group and 28.318 [20.644–32.232] in the LD group to 29.819 [24.937–38.064] and 30.837 [25.382–36.789], respectively (*p* < 0.01), and the increase was significantly higher in the HD group (*p* = 0.01). Hypovitaminosis D was found in most patients (71.2%) initially, and serum VitD levels were still <30.0 ng/mL in 46.2% of the participants at the follow-up. A significant increase in the levels of IL-4, IL-6, IL-17A, IL-22, IL-23 and TNF -α [pg/mL] and a decrease in IL-10 levels were reported during the study (*p* < 0.01). A significant positive correlation was observed between 25(OH)D serum levels and sCD40L (R = 0.33; *p* < 0.05) and TNF-α (R = 0.28; *p* < 0.05), and a significant negative correlation was reported between 25(OH)D and IL-23 (R = −0.32; *p* < 0.01) at the beginning of the study. (4) Conclusions: In RRMS patients, the doses of VitD were probably too low to induce beneficial effects on inflammation. Further studies are warranted to determine the effect of VitD supplementation on inflammatory markers in MS patients.

## 1. Introduction

Multiple sclerosis (MS) is an autoimmune disorder of the central nervous system (CNS) associated with chronic inflammation, demyelination and axonal loss. It is one of the most common causes of disability among young adults and affects about 2.5 million people worldwide, mostly women [1,2,3].

One of the most common hypotheses of MS pathology is associated with the development of the disease and activation of auto-reactive lymphocytes in the secondary lymphoid tissues, as well as their reactivation in the CNS, causing a pro-inflammatory response mediated by Th1-cells and Th17-cells, leading to the damage of myelin and oligodendrocytes. In response to axonal injury, microglial cells are activated and are an important source of cytokines, which mediate inflammatory processes, thus contributing to neurodegeneration [2]. Researchers indicate that the underlying cause of the disease may be associated with an imbalance of pro- and anti-inflammatory cytokines [3]. Sustained cytokine production plays an essential role in the pathogenesis of MS, and cytokine concentrations may reflect the level of inflammation and correlate with the progression of the disease [4]. Considering the above, biomarkers for MS and its pathogenesis are still being investigated. Cytokines are potential candidates that may indicate MS activity and progression. They could also provide information about the pathogenesis of the disease [5].

The etiology of MS is unknown and is believed to result from a specific response of the immune system to self-antigens in genetically predisposed individuals combined with the influence of environmental factors [2]. Furthermore, apart from the geographical latitude, vitamin D (VitD) deficiency is considered to be one of the risk factors. Furthermore, VitD may play a major role in immune regulation in MS [6]. Interestingly, in a study by Oveland et al. using the cuprizone model, which reflects de- and remyelination in MS, it was found that 1,25-dihydroxy vitamin D could have a positive impact on early remyelination by regulating the proteins participating in calcium metabolism and mitochondrial function [7]. Additionally, studies emphasized a modulating effect of VitD on the differentiation of the immune and inflammatory cells and its influence on cytokine secretion, which suggests its essential role in the pathogenesis of chronic inflammatory disorders [8]. 

Chronic inflammation in MS patients may lead to irreversible tissue damage. Therefore, the regulation of such inflammation seems essential [8]. Furthermore, it is assumed that the pro- and anti-inflammatory cytokine imbalance is more noticeable during hypovitaminosis D, which might augment inflammation in MS patients [6,9,10]. Therefore, higher levels of serum VitD are connected not only with a reduction in MS risk but also with lower activity and severity of the disease [8,11,12]. Previous studies have confirmed that the effect of VitD in MS patients depended on the dose and the study duration [13] (800–14,000 IU/day for 8 to 96 weeks) [6,12,14,15,16,17,18,19,20,21,22,23]. Studies have not provided a clear answer about the beneficial effect of VitD supplementation on inflammatory markers. However, it is a common practice in MS patients, which indicates the necessity for developing global recommendations on supplementation and the dose. 

The aim of this study was to assess whether VitD supplementation could improve inflammation in relapsing-remitting multiple sclerosis (RRMS) patients and whether the effect varied depending on the dose. We examined the effects of two doses of oral VitD (2000 IU/day and 15,960 IU/month) on systemic inflammatory markers in the blood serum of 52 patients with RRMS. 

## 2. Materials and Methods

### 2.1. The Study Design and Patient Details

The study group consisted of adult patients diagnosed with MS based on the revised McDonald criteria (2017) [24] and treated at the Multiple Sclerosis Center in Zabrze, Poland. The inclusion criteria were disease-modifying therapy (DMT) for at least six months and residence in the region of Silesia (latitude 49°–50° N). The exclusion criteria were as follows: neurological diseases other than MS, disorders affecting calcium-phosphate homeostasis, disease phenotypes other than RRMS, relapse in the last six months, an Expanded Disability Status Scale (EDSS) score > 5, intake of drugs or a diet that could affect calcium-phosphate metabolism, intake of VitD supplements within six months before the inclusion, traveling to a different climatic zone in the last six months before the enrollment, staying indoors only or working underground, menopausal period, current or planned pregnancy within the next six months and breastfeeding. 

Out of 309 patients, 52 subjects were selected. They met the inclusion and exclusion criteria, gave informed consent to participate and followed a regimen of supplementation. The study group was divided into two subgroups in which 6-month VitD supplementation was introduced:

Low dose (LD)—29 MS patients were given a low dose of VitD (calcifediol—15,960 IU/month; i.e., 530 IU/day);

High dose (HD)—23 MS patients were given a high dose of VitD (cholecalciferol—2000 IU/day; i.e., 60,000 IU/month).

All participants were on DMT such as interferonβ-1b (IFNβ-1b) (*n* = 5), IFNβ-1a (*n* = 5), pegylated IFNβ-1a (*n* = 3), dimethyl fumarate (*n* = 21), teriflunomide (*n* = 8), ocrelizumab (*n* = 6), fingolimod (*n* = 2) and cladribine (*n* = 2).

### 2.2. Serum Measurements 

Blood samples were collected on the day of inclusion (from October 2021 to March 2022) and after six months of VitD supplementation. The serum samples were collected and stored at −80 °C until analysis. All laboratory tests were performed at baseline and at the end of the study in accordance with the manufacturer’s protocol. The obtained results were compared with the applicable standards for specific test methods. 

A quantitative determination of 25(OH)D in the blood serum was performed at the Silesia LabMed Research and Implementation Center, Medical University of Silesia, Poland, using a sandwich enzyme-linked immunosorbent assay (ELISA) kit (IDK^®^ 25-OH-Vitamin D ELISA, Immuniq, Żory, Poland). VitD sufficiency was defined as 25(OH)D serum level ≥30.0 ng/mL and insufficiency as 20.0–29.9 ng/mL, whereas deficiency was defined as <20.0 ng/mL and severe deficiency as <10.0 ng/mL [25]. Hypovitaminosis D was associated with a concentration <30.0 ng/mL (75.0 nmol/l) [26,27]. 

The concentrations of selected cytokines involved in the Th17 immune response pathway, such as IL-4, IL-6, IL-10, IL-17A, IL-22, IL-23, TNF-α and sCD40L, were determined simultaneously using multiplex bead immunoassays (Bio-Plex^®^ 200 system, Bio-Rad Laboratories, Inc., Hercules, CA, USA). Each sample was incubated with antibody-coupled beads, biotinylated secondary antibodies and streptavidin–phycoerythrin. The beads were read on a Luminex System (Bioplex 200, Bio-Rad), and the data were analyzed using Bioplex Manager Software, version 6.1.1 (Bio-Rad Laboratories, Hercules, CA, USA). All cytokine analyses were performed at the Department of Microbiology and Immunology, Faculty of Medical Sciences, in Zabrze, Medical University of Silesia, Katowice, Poland. 

### 2.3. Statistical Analysis

Data are shown as median with the quartile range. To compare quantitative variables, the Wilcoxon test for dependent measurements was introduced. Spearman’s Rho coefficient was used to estimate correlations. The analysis was conducted using the R language in the RStudio (Posit, Boston, MA, USA) environment. 

The relationship between the dose of VitD supplementation and changes in the selected cytokines over time was determined using the nparLD test to calculate *p* values for the time effect, group effect and the interaction between group effect and time. Statistical significance was defined as *p* < 0.05. 

The GEE model with adjustment for the BMI and age was performed to confirm the obtained results (Supplementary Materials).

## 3. Results

A total of 52 patients who fulfilled the inclusion and exclusion criteria and supplemented VitD during the study period constituted a study group (23 [44.23%] were given an HD of VitD and 29 [55.77%] an LD). 

Most patients were females [63.46%]. The median age [years] was 39.5 [34.5–49.8] in the HD group and 47 [40.0–55.0] in the LD group, and a significant difference was observed between both groups (*p* = 0.028). Significant differences in body weight [kg] (74.0 [65.0–89.0] in the HD group and 65.0 [62.0–75.0] in the LD group) and height [m] were also observed between the HD group and the LD group (*p* = 0.014 and *p* = 0.001, respectively). Considering the BMI [kg/m^2^] (23.6 [22.8–27.1] in the HD group and 23.4 [22.1–26.8] in the LD group), no statistical differences were observed (*p* = 0.496). Obesity (BMI ≥ 25 kg/m^2^) was present in 34.78% of patients in the HD group and 31.03% of patients in the LD group. Of all participants, 28.85% reported smoking cigarettes, and 17.31% reported regular physical activity. Basic demographic data and disease characteristics are given in Table 1.

**Figure 1:**
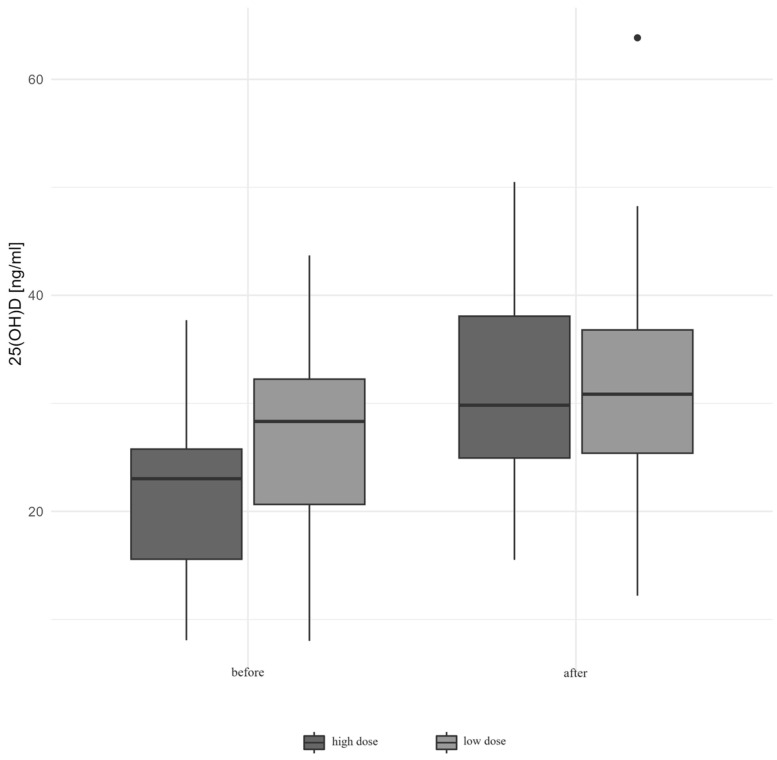
Concentrations of vitamin D before and after six months of vitamin D supplementation depending on the supplemented dose.

At the beginning of the study, the median 25(OH)D concentration [ng/mL] was 23.023 [15.578–25.76] in the HD group and 28.318 [20.644–32.232] in the LD group. At the follow-up, the level increased significantly to 29.819 [24.937–38.064] and 30.837 [25.382–36.789], respectively (*p* < 0.01). The increase in 25(OH)D was significantly higher in the HD group (*p* = 0.01), and, after six months of supplementation, the median concentration was similar in both groups. Therefore, the dynamics of VitD increase was significantly higher in the HD group (*p* = 0.01). The median serum 25(OH)D level is given in Table 2. A change in the 25(OH)D serum level depending on the supplemented dose is presented in Figure 1. 

Hypovitaminosis D was found in many participants initially (71.2%) and at the end of the study. VitD levels were still insufficient in 46.2% of the whole study group. The VitD status of the participants based on the 25(OH)D serum level is given in Table 3.

The median EDSS score (3.0 [2.5–3.5] in HD and LD groups) did not change significantly after VitD supplementation in both groups (*p* = 0.709).

**Figure 2:**
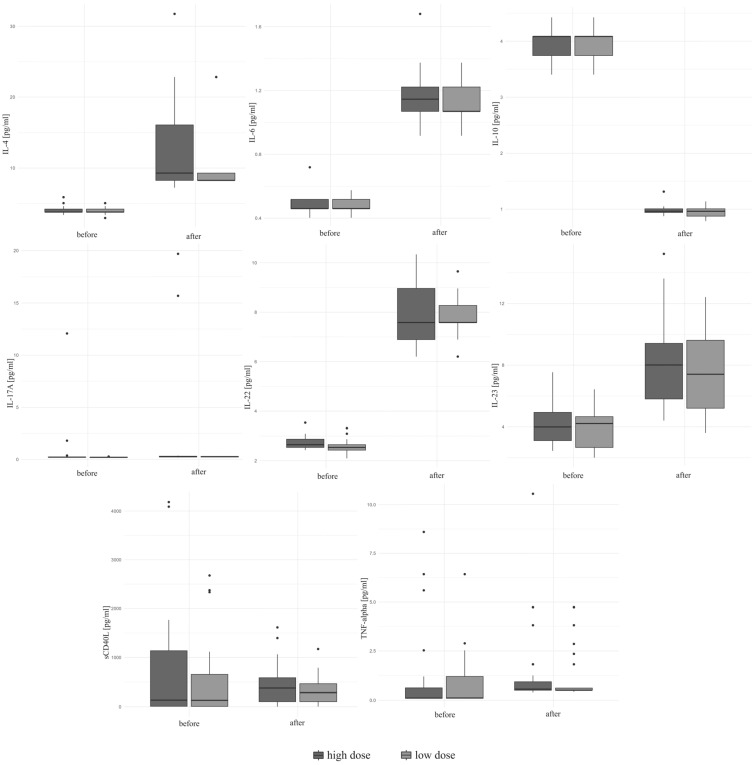
The concentrations of selected cytokines before and after six months of vitamin D supplementation depending on the supplemented dose.

A significant increase in IL-4, IL-6, IL-17A, IL-22, IL-23 and TNF-α was observed during the study (*p* < 0.01 for all markers) regardless of the dose of VitD (*p* > 0.05). A statistically significant decrease in IL-10 levels was observed during the study (*p* < 0.01) irrespective of the dose of VitD. An increase in sCD40L concentrations was noted during the study, but it was not statistically significant (*p* = 0.79). The concentrations of selected cytokines and sCD40L at the beginning of the study and after six months of VitD supplementation are given in Table 4. A change in the serum level of selected cytokines depending on the supplemented dose is given in Figure 2.

A significant positive correlation was found between serum concentrations of 25(OH)D and sCD40L (R = 0.33; *p* < 0.05) and TNF-α (R = 0.28; *p* < 0.05), and a significant negative correlation was observed between serum levels of 25(OH)D and IL-23 (R = −0.32; *p* < 0.01), but only at the study onset (Table 5). 

## 4. Discussion

Significant differences in age, body weight and height between both study groups did not affect the results. According to some studies, age-related changes in the metabolism of VitD are most pronounced with advancing age [28], and, considering the BMI, no statistically significant differences were found between both groups. 

IL-4, which is an anti-inflammatory cytokine, has a crucial role in the pathogenesis of MS. It is produced mainly by Th2-cells, and its early secretion may lead to the differentiation of Th-cells towards the Th2 phenotype. Furthermore, it may inhibit the expression and release of pro-inflammatory cytokines, such as IL-1, TNF-α, IL-6 or IL-8 [29]. According to recent studies, serum levels of IL-4 are elevated in RRMS patients compared to healthy controls [29,30]. However, other authors reported opposite results. Bartosik-Psujek et al. showed that significantly lower serum levels of IL-4 were found during remission and relapses compared to the healthy controls [31]. Furthermore, IL-4 concentrations were higher in patients with mild disability (depending on the EDSS score) compared to those with moderate and severe disability [30]. Burton et al. indicated that a high dose of VitD supplementation (about 10 000 IU/day) for 52 weeks in MS patients resulted in a decrease in T-cell reactivity and proliferation, which was the most evident in patients with 25(OH)D concentrations ≥100 nmol/L at the follow-up. However, they did not observe any significant changes in the cytokine profiles, including IL-4, IFN-γ, IL-1β, IL-2, IL-5, IL-6, IL-10, IL-12 or IL-13 [16]. Similarly, Jorde et al. evaluated the effect of a one-year supplementation with 40,000 IU and 20,000 IU of VitD per week in overweight and obese subjects. They found no statistically significant relationships between serum concentrations of IL-4, IL-10, IL-17, IL-2, IL-5, IL-12, IL-13, IFN-γ and 25(OH)D at baseline [32]. 

IL-10 is another anti-inflammatory cytokine that also participates in the immune response and is synthesized by CD4+ Th2-cells, monocytes and B-cells. It inhibits Th1-type cytokines, such as IL-2 and IFN-γ, and can deactivate monocytes and macrophages, suppressing the induced pro-inflammatory cytokine production (TNF-α, IL-1, IL-6, IL-8 and IL-12). IL-10 can promote the survival of nervous tissue [33]. Importantly, studies have demonstrated that serum IL-10 production is inversely correlated with the number of lesions and clinical disability [34,35]. Additionally, serum concentrations of IL-10 are reported to be a risk factor for other relapses in individuals with clinically isolated syndrome (CIS) [34]. Furthermore, Kallaur et al. found that IL-10 concentrations were higher in inactive RRMS compared to patients with the active disease [30]. IL-10 can be induced by VitD and therefore can reduce inflammation by suppressing T-cell activation [9]. Mosayebi et al. evaluated the influence of a six-month intramuscular supplementation with 300,000 IU/month of VitD or placebo on the concentrations of selected cytokines in 62 MS patients. They observed a significant increase in the levels of IL-10 and TGF-β in the intervention group, whereas IFN-γ concentrations remained unchanged [14]. Similarly, in their 12-week trial with RRMS patients, Ashtari et al. assessed the effect of 50,000 IU of VitD every five days and reported a significant increase in IL-10 levels [15], which is in line with other studies [6]. Additionally, Walawska-Hrycek et al. reported an increase in IL-10, TGF-β and IFN-γ with the increase in serum VitD levels, whereas no change was reported in the case of IL-17 [17]. As in our study, they introduced low doses of VitD for one year in VitD-deficient RRMS patients, adjusting the dose to VitD serum concentrations (1000 IU/day, 500 IU daily or no supplementation). However, in our study, we included patients with both hypovitaminosis and normal serum VitD levels, which may be responsible for the differences in the results. Similar to our observations, Fujita et al. and Nino et al. indicated that VitD could downregulate the production of IL-10 [9]. 

IL-6, which is a pro-inflammatory cytokine, participates in the activation, growth and differentiation of T-cells. Neuroinflammation is associated with the upregulation of IL-6 leading to neuronal damage, and in the presence of TGF-β, it may promote the differentiation of T-cells into Th17-cells, thus increasing the secretion of IL-17A and IL-22. IL-6 levels are higher in RRMS patients than in healthy controls [30]. Furthermore, there is a correlation between increased IL-6 levels in the cerebrospinal fluid (CSF) and an increased relapse rate and disability [36]. In our study, the increase in IL-6 may be subsequent to elevated IL-17A levels. Furthermore, it may be associated with a decreased expression of IL-10 [9]. In healthy subjects deficient in VitD, Fassio et al. noticed a decrease in serum levels of IL-6 and IL-17A with no clear relationship to the supplementation period (4, 8, 12 or 16 weeks) or the dose (10,000 IU/day of VitD for eight weeks or 1000 IU/day for four weeks; 50,000 IU/week for 12 weeks and 100,000 IU every other week for 12 weeks). They observed no significant changes in other parameters, including IL-23, IL-8, IL-10 or TNFα [37].

However, pro-inflammatory IL-23 is responsible for maintaining and augmenting the activation of Th17-cells. In the presence of IL-6 and TGF-β, naive CD4+ T-cells may differentiate into Th17-lymphocytes, which are less pathogenic [38]. IL-23 levels tend to be significantly higher in MS patients compared to healthy subjects [39]. Additionally, in our study, we observed a significant negative correlation between the concentrations of VitD and IL-23. Hong et al. also reported that serum 25(OH)D and IL-23 levels were inversely correlated in patients with rheumatoid arthritis, which is also associated with chronic inflammation [40]. 

The above pro-inflammatory IL-17 is produced by Th17 cells, which are important inflammatory mediators in MS and may disrupt the brain–blood barrier (BBB) [34]. This cytokine enhances the production of IL-6, TNF-α, IL-1 and reactive oxygen species, stimulating neuroinflammation [9,41]. IL-17 is highly expressed in MS lesions [20], and its concentration is significantly higher in MS patients than in healthy controls [38,39,42,43]. Similar to IL-6, IL-17 is probably related to the disability level [44], and, according to recent studies, the level of IL-17A is associated with MRI disease activity. A study found that Th17-cells correlated with strongly neurodegenerative (T1-hypointense) lesions in MS [34]. Kallaur et al. showed that IL-17 level was associated with the disease activity. It was higher during inactive RRMS compared to the active disease [30]. Studies have demonstrated that VitD may inhibit the expression of IL-17 in human lymphocytes T via the VitD receptor (VDR), thus preventing autoimmunity [43,45]. In a study on 40 RRMS patients randomized to receive 10,400 IU or 800 IU of cholecalciferol for six months, Sotirchos et al. indicated that high-dose VitD supplementation induced a reduction in the production of IL-17 by CD4(+) T-cells. However, they found no differences in the concentrations of 51 cytokines in both groups (including IL-17, IL-1A, IL-1RA, IL-1β, IL-2, IL-4, IL-5, IL-6, IL-7, IL-8, IL-10 IL-13, IL-15, TNF-α, IFN-β, IFN-γ and CD40L) [12]. On the other hand, 50,000 IU of VitD every five days for 12 weeks in a group of 94 patients with RRMS contributed to a significant improvement in the concentration of IL-17 compared to the placebo group adjusted by EDSS scores [19]. Hashemi et al. also noticed that treatment with VitD (50 000 IU every seven days for eight weeks) in patients with MS increased the concentrations of anti-inflammatory cytokines, such as IL-27, TGF-β1 and IL-10, while the levels of pro-inflammatory interleukins, such as IL-17A and IL-6, were decreased [6]. They reported that the concentrations of IL-17A increased in the pro-inflammatory state due to the deficiency level of VitD [6]. Similar results to those in our study were obtained by Golan et al., who introduced 800 IU/d or 4370 IU/d of VitD for one year in RRMS patients and observed an increase in the level of IL-17 in patients receiving a low dose. However, high-dose supplementation caused a heterogeneous response [20]. In contrast, as indicated in the study by Walawska-Hrycek et al., no changes in the concentrations of IL-17 were found after low-dose VitD supplementation [17]. 

It is suggested that the pro-inflammatory IL-22 is also involved in the pathogenesis of MS [46]. It is a Th17-linked cytokine that is presumed to participate in the impairment of the BBB, which allows the infiltration of lymphocytes, thus contributing to the severity of the disease [42,46]. Serum levels of IL-22 were significantly higher in all relapsing-remitting and progressive MS patients compared to healthy individuals [39,46]. It is reported that VitD decreases the proliferation of CD4+ T-cells and B-cells, thus reducing the population of cells producing IL-22 [47]. In their study, Aivo et al. used the supplementation of 20,000 IU of cholecalciferol weekly in MS patients treated with IFNβ-1b for one year, which resulted in an increase in TGF-β levels in the intervention group without affecting the levels of IL-22, INF-γ, IL-17A, IL-2, IL-10, IL-9, IL-6, IL-13, IL-4, IL-5, IL-1β or TNF-α in either group. Additionally, they noted an increase in IFN- γ, IL-17 and IL-9 concentrations, which is not in line with other scientific reports. However, the increase was not statistically significant [21]. 

TNF-α is a pleiotropic pro-inflammatory cytokine produced by macrophages, monocytes and differentiated T cells and is associated with a Th1 response [42] and demyelination [4]. The expression of TNF-α is upregulated in MS patients [48], and its concentration correlates with disease severity and progression [49]. TNF-α levels were higher in patients with inactive RRMS compared to those with the active disease [30]. In the study by Mahon et al., 6-month VitD supplementation (1000 IU/d) increased TGF-β1 levels, whereas no changes were observed in the concentrations of IFN-γ, TNF-α and IL-13. However, the supplementation increased 25(OH)D serum levels [23]. Similar results concerning TNF-α were observed in other studies [12,21,37,50]. A significant positive correlation was found between serum TNF-α and VitD levels, which was also noticed in a study on patients with chronic obstructive pulmonary disease [51]. However, a negative correlation was also reported [52]. 

Additionally, serum levels of sCD40L were positively correlated with the 25(OH)D concentrations in our study. This marker is postulated to play a crucial role in the pathogenesis of MS and is responsible for the impairment of the BBB [53,54]. It is associated with disease progression [55]. Interactions between CD40 and its ligand are proposed to induce pro-inflammatory cytokines such as TNF-α [56]. sCD40L levels are higher in the CSF and serum of MS patients compared to healthy controls, and its levels are correlated with the disability level [53,54,55]. Interestingly, in patients with autoimmune hepatitis (AIH), the percentage of free VitD (free 25(OH)D/total 25(OH)D) in patients with AIH was negatively correlated with sCD40L concentrations [57]. 

During our study, the levels of pro-inflammatory cytokines increased and a heterogenous response was observed in the case of anti-inflammatory markers. The doses of VitD in both study groups might be too low to induce beneficial results. The trend towards decreased concentrations of pro-inflammatory cytokines was observed in studies with higher doses of VitD [12,14,15,20], which confirmed its possible desirable effect. The variance of the obtained results may also be related to the duration of supplementation or the sample size. Additionally, a low dose of vitamin D as a comparator could reduce the ability to notice minor differences compared to placebo. Furthermore, various studies on patients with different initial VitD statuses showed that those deficient in VitD (<50 nmol/L) could be more prone to immune changes after supplementation [58]. These reports could be supported by our observations that correlations between VitD and selected inflammatory markers were significant only at the beginning of the study. Additionally, differences in genetic inheritance and VDR polymorphisms in the general population should also be considered [9]. Also, a different cytokine measurement method (ELISA) and the use of peripheral blood mononuclear cells (PBMCs) in the analysis might have contributed to the differences obtained by other researchers [59]. In our study, the changes in the cytokine balance may be associated with confounding factors other than VitD, which is difficult to determine. Baseline serum 25(OH)D concentrations were assessed during the autumn-winter period, whereas the level at the follow-up was established during the spring-summer time, and seasonal variation in immune cell subsets and cytokines has been previously reported [20]. Also, during infections, especially during the COVID-19 outbreak [60], or in various conditions associated with immune dysregulation, there are changes in pro- and anti-inflammatory homeostasis. Additionally, in RRMS, even during remission, there is a complex system of pro- and anti-inflammatory mediators that affect each other to modulate the progression and activity of the disease [30]. Changes in cytokine homeostasis may also be associated with the disease duration. Also, different DMTs could be responsible for the effects, which can be related to different immunomodulatory effects of therapeutic agents [61]. 

## 5. Conclusions

There is emerging evidence that VitD may be a potential immunomodulator due to its ability to reduce inflammation. However, researchers have inconsistent conclusions on its influence on the cytokine balance in MS patients, and studies vary in terms of the supplementation dose. Low-dose VitD supplementation may be sufficient to normalize serum 25(OH)D levels, although the doses might be too low to induce anti-inflammatory effects. The elevation of pro-inflammatory cytokines and IL-4 and the decrease in IL-10 levels may be associated with different clinical conditions and confounding factors. Inflammatory mediators are differently expressed during the disease course, which may suggest that various environmental and individual factors are involved in the changes in the immune balance and clinical presentation of the disease. However, considering the existence of favorable correlations between selected inflammatory markers, a beneficial role of VitD on the immune balance in patients with RRMS cannot be ruled out.

Further studies are warranted to assess the necessity of VitD supplementation in MS patients and to establish a supplemented dose and optimal serum 25(OH)D levels necessary to reduce inflammation.

## 6. Limitations

Our study is limited to a small number of participants and the lack of healthy controls. The study was not placebo-controlled. Furthermore, we included patients treated with different DMTs, which may be the reason for the heterogeneous immunological response to VitD supplementation. However, other studies do not support this observation, and no scientific reports show that DMT treatment could affect the VDR receptor by which VitD exerts its action. 

## Figures and Tables

**Table 1:** Basic characteristics of the study group depending on the dose of VitD supplementation.

	HD ^1^			LD ^2^			*p* Value
Variable	Median	q1	q3	Median	q1	q3	
Age [years]	39.5	34.5	49.8	47.0	40.0	55.0	0.028
Weight [kg]	74.0	65.0	89.0	65.0	62.0	75.0	0.014
Height [m]	1.8	1.7	1.8	1.7	1.6	1.8	0.001
BMI [kg/m^2^]	23.6	22.8	27.1	23.4	22.1	26.8	0.496
Age at diagnosis [years]	34.0	26.0	47.0	33.5	28.5	43.8	0.626
Disease duration [years]	6.0	4.0	12.0	8.0	3.0	15.8	0.215
Age at first symptoms [years]	31.0	24.5	43.0	32.0	27.5	39.3	0.601
Duration of immunomodulatory treatment [months]	53.0	42.0	104.0	80.5	41.3	132.8	0.866
The number of MS ^3^ relapses	2.0	1.0	4.0	3.0	1.0	5.3	0.240
Time from the last relapse [months]	56.0	31.5	70.0	55.0	33.5	82.0	0.724
The number of relapses treated with GCs ^4^	2.0	1.0	3.5	2.0	1.0	5.5	0.125
Time from the last administration of GCs [months]	58.0	37.0	77.0	55.0	31.5	74.5	0.652
EDSS ^5^ [score]	3.0	2.5	3.5	3.0	2.5	3.5	0.746
Time from smoking initiation [years]	20.0	10.0	27.5	20.0	18.8	27.5	0.396
The number of cigarettes/day	15.0	11.3	18.8	12.5	10.0	18.8	0.622
Amount of physical activity [min/week]	165.0	67.5	292.5	120.0	60.0	210.0	0.368

^1^ High dose of supplemental vitamin D, ^2^ low dose of supplemental vitamin D, ^3^ multiple sclerosis, ^4^ Glucocorticosteroids, ^5^ Expanded Disability Status Scale.

**Table 2:** Serum concentration of 25(OH)D before and after vitamin D supplementation.

		Before			After			GE ^4^ (p)	TE ^5^ (p)	GETE ^6^ (p)
Variable	Group	Median	q1	q3	Median	q1	q3			
25(OH)D ^1^ [ng/mL]	HD ^2^	23.023	15.578	25.760	29.819	24.937	38.064	0.19	0.00	0.01
	LD ^3^	28.318	20.644	32.232	30.837	25.382	36.789			

^1^ 25-Hydroxyvitamin D, ^2^ high dose of supplemented vitamin D, ^3^ low dose of supplemented vitamin D, ^4^ group effect, ^5^ time effect, ^6^ group effect*time effect.

**Table 3:** The vitamin D status of the study participants depending on 25(OH)D serum levels before and after six months of supplementation.

Before	After
Severe deficiency—5.8%	Severe deficiency–0%
Deficiency—23.1% Insufficiency—42.3% Sufficiency—28.8% High concentration—0%	Deficiency—11.5% Insufficiency—34.6% Sufficiency—50% High concentration—3.9%
Hypovitaminosis D—71.2%	Hypovitaminosis D—46.2%

**Table 4:** Serum concentrations of selected cytokines and sCD40L before and after vitamin D supplementation.

		Before			After			GE ^6^ (*p*)	TE ^7^ (p)	GETE ^8^ (p)
Variable	Group	Median	q1	q3	Median	q1	q3			
IL-4 ^3^ [pg/mL]	HD ^1^	3.780	3.780	4.200	9.290	8.258	16.060	0.16	<0.001	0.58
	LD ^2^	3.780	3.780	4.200	8.258	8.258	9.290			
IL-10 [pg/mL]	HD	4.085	3.744	4.085	0.964	0.942	1.008	0.68	0.00	0.86
	LD	4.085	3.744	4.085	0.964	0.876	1.008			
IL-6 [pg/mL]	HD	0.460	0.460	0.518	1.145	1.069	1.222	0.85	<0.001	0.56
	LD	0.460	0.460	0.518	1.069	1.069	1.222			
IL-17A [pg/mL]	HD	0.221	0.206	0.243	0.267	0.232	0.312	0.10	<0.001	0.06
	LD	0.206	0.191	0.221	0.267	0.232	0.285			
IL-22 [pg/mL]	HD	2.651	2.540	2.872	7.583	6.893	8.961	0.15	<0.001	0.08
	LD	2.540	2.430	2.651	7.583	7.583	8.272			
IL-23 [pg/mL]	HD	3.993	3.106	4.936	8.011	5.808	9.413	0.52	<0.001	0.61
	LD	4.215	2.662	4.658	7.410	5.207	9.613			
sCD40L ^4^ [pg/mL]	HD	134.510	10.287	1136.120	376.690	104.245	585.620	0.35	0.79	0.87
	LD	130.980	8.747	654.260	285.640	104.440	464.630			
TNF-α ^5^ [pg/mL]	HD	0.110	0.100	0.620	0.555	0.499	0.93	0.94	<0.001	0.53
	LD	0.115	0.100	1.200	0.499	0.499	0.61			

^1^ High dose of supplemented vitamin D, ^2^ low dose of supplemented vitamin D, ^3^ Interleukin, ^4^ soluble CD40 ligand, ^5^ Tumor necrosis factor α, ^6^ group effect, ^7^ time effect, ^8^ group effect*time effect.

**Table 5:** Correlations between 25(OH)D serum levels and concentrations of selected cytokines and sCD40L before vitamin D supplementation.

Time	Variable 1	Variable 2	R	*p* Value
Before	25(OH)D	sCD40L ^1^	0.33	0.01700
		IL-23 ^2^	−0.32	0.00536
		TNF-α ^3^	0.28	0.04490

^1^ Soluble CD40 ligand, ^2^ Interleukin-23, ^3^ Tumor necrosis factor α.

## Data Availability

The dataset obtained in the research is available from the corresponding author upon a reasonable request due to privacy of participants.

## Supplementary Materials

The following supporting information can be downloaded at: https://www.mdpi.com/article/10.3390/biomedicines12071580/s1. A file with the GEE model with adjustment for the BMI and age can be downloaded, File S1: GEE model.
