# Supplementary material for: Can Vitamin D Supplementation Improve Inflammation in Relapsing-Remitting Multiple Sclerosis Patients?
Source: Biomedicines. 2024 Jul 17;12(7):1580. doi: 10.3390/biomedicines12071580 (PMC11274703; doi:10.3390/biomedicines12071580)
Supplement: Supplementary file 1 [file biomedicines-12-01580-s001.zip › biomedicines-3040348-supplementary.pdf]

```
> lapply(gees2, summary)
[[1]]

Call:
geeglm(formula = `25OHD` ~ Time + Supplemented_dose + BMI,
    data = ., id = Lp)

 Coefficients:
                  Estimate Std.err  Wald Pr(>|W|)
(Intercept)         15.414   3.541 18.95  1.3e-05 ***
Time                 6.635   1.788 13.78  0.00021 ***
Supplemented_dose    3.030   1.783  2.89  0.08915 .
BMI                  0.031   0.103  0.09  0.76279
---
Signif. codes:  0 ‘***’ 0.001 ‘**’ 0.01 ‘*’ 0.05 ‘.’ 0.1 ‘ ’ 1

Correlation structure = independence
Estimated Scale Parameters:

            Estimate Std.err
(Intercept)     83.1    12.6
Number of clusters:   104  Maximum cluster size: 1

[[2]]

Call:
geeglm(formula = IL_4 ~ Time + Supplemented_dose + BMI, data = .,
    id = Lp)

 Coefficients:
                  Estimate Std.err  Wald Pr(>|W|)
```

```
(Intercept)        -5.8634  2.3045  6.47    0.011 *
Time              7.3413  0.7870 87.02   <2e-16 ***
Supplemented_dose    -1.5211  0.8396  3.28    0.070 .
BMI               0.1297  0.0806  2.59    0.107
---
Signif. codes:  0 ‘***’ 0.001 ‘**’ 0.01 ‘*’ 0.05 ‘.’ 0.1 ‘ ’ 1

Correlation structure = independence
Estimated Scale Parameters:

           Estimate Std.err
(Intercept)     16.1    4.48
Number of clusters:   104  Maximum cluster size: 1

[[3]]

Call:
geeglm(formula = IL_10 ~ Time + Supplemented_dose + BMI, data = .,
    id = Lp)

 Coefficients:
                  Estimate Std.err    Wald Pr(>|W|)
(Intercept)         6.8345  0.1031 4394.69   <2e-16 ***
Time             -2.9879  0.0362 6806.91   <2e-16 ***
Supplemented_dose    -0.0105  0.0369    0.08     0.78
BMI               0.0044  0.0027    2.65     0.10
---
Signif. codes:  0 ‘***’ 0.001 ‘**’ 0.01 ‘*’ 0.05 ‘.’ 0.1 ‘ ’ 1

Correlation structure = independence
Estimated Scale Parameters:
```

```
          Estimate Std.err
(Intercept)   0.0341 0.00629
Number of clusters:   104  Maximum cluster size: 1 

[[4]]

Call:
geeglm(formula = IL_6 ~ Time + Supplemented_dose + BMI, data = .,
   id = Lp)

 Coefficients:
                  Estimate  Std.err    Wald Pr(>|W|)
(Intercept)       -0.23946  0.04651   26.51  2.6e-07 ***
Time               0.66427  0.01976 1129.85  < 2e-16 ***
Supplemented_dose -0.02317  0.02059    1.27     0.26
BMI                0.00259  0.00157    2.71     0.10 .
---
Signif. codes:  0 ‘***’ 0.001 ‘**’ 0.01 ‘*’ 0.05 ‘.’ 0.1 ‘ ’ 1

Correlation structure = independence 
Estimated Scale Parameters:

          Estimate Std.err
(Intercept)   0.0102 0.00273
Number of clusters:   104  Maximum cluster size: 1 

[[5]]

Call:
geeglm(formula = IL_17A ~ Time + Supplemented_dose + BMI, data = .,
```

```
   id = Lp)

 Coefficients:
                  Estimate   Std.err Wald Pr(>|W|)
(Intercept)         0.589316  0.601284 0.96    0.327
Time               0.462659  0.509871 0.82    0.364
Supplemented_dose   -1.061609  0.576135 3.40    0.065 .
BMI                0.000508  0.008175 0.00    0.950
---
Signif. codes:  0 '***' 0.001 '**' 0.01 '*' 0.05 '.' 0.1 ' ' 1

Correlation structure = independence
Estimated Scale Parameters:

           Estimate Std.err
(Intercept)    6.76    3.84
Number of clusters:   104  Maximum cluster size: 1

[[6]]

Call:
geeglm(formula = IL_22 ~ Time + Supplemented_dose + BMI, data = .,
   id = Lp)

 Coefficients:
                 Estimate  Std.err    Wald Pr(>|W|)
(Intercept)        -2.40654  0.22838  111.04   <2e-16 ***
Time               5.17062  0.14101 1344.53   <2e-16 ***
Supplemented_dose   -0.13038  0.14753    0.78     0.38
BMI              -0.00159  0.00736    0.05     0.83
---
```

Signif. codes: 0 ‘***’ 0.001 ‘**’ 0.01 ‘*’ 0.05 ‘.’ 0.1 ‘ ’ 1

Correlation structure = independence

Estimated Scale Parameters:

```
            Estimate Std.err
(Intercept)    0.517  0.0947
```

Number of clusters: 104 Maximum cluster size: 1

[[7]]

Call:

```
geeglm(formula = IL_23 ~ Time + Supplemented_dose + Age_years,
    data = ., id = Lp)
```

Coefficients:

```
                  Estimate Std.err  Wald Pr(>|W|)
(Intercept)        -0.3901  0.9244  0.18     0.67
Time                3.6882  0.4217 76.49   <2e-16 ***
Supplemented_dose  -0.5156  0.4449  1.34     0.25
Age_years           0.0231  0.0188  1.52     0.22
---
```

Signif. codes: 0 ‘***’ 0.001 ‘**’ 0.01 ‘*’ 0.05 ‘.’ 0.1 ‘ ’ 1

Correlation structure = independence

Estimated Scale Parameters:

```
            Estimate Std.err
(Intercept)     4.54   0.727
```

Number of clusters: 102 Maximum cluster size: 1

```
[[8]]

Call:
geeglm(formula = sCD40L ~ Time + Supplemented_dose + Age_years,
   data = ., id = Lp)

 Coefficients:
                 Estimate Std.err Wald Pr(>|W|)
(Intercept)         692.05  306.98 5.08    0.024 *
Time               -270.21  143.04 3.57    0.059 .
Supplemented_dose    -264.58  175.23 2.28    0.131
Age_years             8.15    8.42 0.94    0.333
---
Signif. codes:  0 ‘***’ 0.001 ‘**’ 0.01 ‘*’ 0.05 ‘.’ 0.1 ‘ ’ 1

Correlation structure = independence
Estimated Scale Parameters:

          Estimate Std.err
(Intercept)   521725  152955
Number of clusters:   102  Maximum cluster size: 1

[[9]]

Call:
geeglm(formula = TNF_α ~ Time + Supplemented_dose + Age_years,
   data = ., id = Lp)

 Coefficients:
                 Estimate Std.err Wald Pr(>|W|)
(Intercept)         -0.134   0.764 0.03     0.86
```

```
Time                 0.240   0.356 0.46     0.50
Supplemented_dose     -0.483   0.420 1.33     0.25
Age_years            0.026   0.018 2.07     0.15

Correlation structure = independence
Estimated Scale Parameters:

            Estimate Std.err
(Intercept)     3.23   0.991
Number of clusters:   102  Maximum cluster size: 1

> lapply(gees3, summary)
[[1]]

Call:
geeglm(formula = `25OHD` ~ Time + Supplemented_dose + Age_years,
    data = ., id = Lp)

 Coefficients:
                  Estimate Std.err  Wald Pr(>|W|)
(Intercept)         11.7035  4.6411  6.36  0.01168 *
Time               6.6119  1.7872 13.69  0.00022 ***
Supplemented_dose     3.0862  1.7644  3.06  0.08026 .
Age_years           0.0950  0.0851  1.25  0.26383
---
Signif. codes:  0 '***' 0.001 '**' 0.01 '*' 0.05 '.' 0.1 ' ' 1

Correlation structure = independence
Estimated Scale Parameters:

            Estimate Std.err
```

```
(Intercept)     81.4    12.8
Number of clusters:   102  Maximum cluster size: 1


[[2]]


Call:
geeglm(formula = IL_4 ~ Time + Supplemented_dose + Age_years,
    data = ., id = Lp)


 Coefficients:
                  Estimate Std.err  Wald Pr(>|W|)
(Intercept)         -4.4510  1.9631  5.14    0.023 *
Time                7.3772  0.8131 82.32   <2e-16 ***
Supplemented_dose    -1.7016  0.9010  3.57    0.059 .
Age_years            0.0442  0.0379  1.36    0.243
---
Signif. codes:  0 '***' 0.001 '**' 0.01 '*' 0.05 '.' 0.1 ' ' 1


Correlation structure = independence
Estimated Scale Parameters:


           Estimate Std.err
(Intercept)     16.9    4.77
Number of clusters:   102  Maximum cluster size: 1


[[3]]


Call:
geeglm(formula = IL_10 ~ Time + Supplemented_dose + Age_years,
    data = ., id = Lp)
```

```
 Coefficients:
                   Estimate   Std.err    Wald Pr(>|W|)
(Intercept)         6.928539  0.097821 5016.76   <2e-16 ***
Time              -2.991969  0.037118 6497.41   <2e-16 ***
Supplemented_dose   -0.014721  0.040646    0.13     0.72
Age-Years           0.000654  0.001431    0.21     0.65
---
Signif. codes:  0 '***' 0.001 '**' 0.01 '*' 0.05 '.' 0.1 ' ' 1

Correlation structure = independence
Estimated Scale Parameters:

           Estimate Std.err
(Intercept)   0.0351 0.00682
Number of clusters:   102  Maximum cluster size: 1

[[4]]

Call:
geeglm(formula = IL_6 ~ Time + Supplemented_dose + Age_years,
    data = ., id = Lp)

 Coefficients:
                   Estimate   Std.err    Wald Pr(>|W|)
(Intercept)        -1.75e-01  5.18e-02   11.44  0.00072 ***
Time               6.66e-01  2.04e-02 1071.11  < 2e-16 ***
Supplemented_dose   -2.21e-02  2.14e-02    1.07  0.30141
Age_years          -2.38e-05  8.25e-04    0.00  0.97695
---
Signif. codes:  0 '***' 0.001 '**' 0.01 '*' 0.05 '.' 0.1 ' ' 1
```

```
Correlation structure = independence
Estimated Scale Parameters:

            Estimate Std.err
(Intercept)   0.0106 0.00293
Number of clusters:   102  Maximum cluster size: 1

[[5]]

Call:
geeglm(formula = IL_17A ~ Time + Suplementowal_dawke + Wiek_lata,
    data = ., id = Lp)

 Coefficients:
                    Estimate Std.err Wald Pr(>|W|)
(Intercept)          2.9819  1.4852 4.03    0.045 *
Time                 0.4713  0.5053 0.87    0.351
Supplemented_dose   -0.8540  0.4630 3.40    0.065 .
Age_years           -0.0550  0.0284 3.74    0.053 .
---
Signif. codes:  0 ‘***’ 0.001 ‘**’ 0.01 ‘*’ 0.05 ‘.’ 0.1 ‘ ’ 1

Correlation structure = independence
Estimated Scale Parameters:

            Estimate Std.err
(Intercept)     6.51    3.56
Number of clusters:   102  Maximum cluster size: 1

[[6]]
```

```
Call:
geeglm(formula = IL_22 ~ Time + Supplemented_dose + Age_years,
    data = ., id = Lp)

 Coefficients:
                  Estimate  Std.err    Wald Pr(>|W|)
(Intercept)        -2.84034  0.32618   75.83   <2e-16 ***
Time               5.16611  0.14222 1319.52   <2e-16 ***
Supplemented_dose   -0.16244  0.14873    1.19     0.27
Age_years           0.00912  0.00612    2.22     0.14
---
Signif. codes:  0 ‘***’ 0.001 ‘**’ 0.01 ‘*’ 0.05 ‘.’ 0.1 ‘ ’ 1

Correlation structure = independence
Estimated Scale Parameters:

            Estimate Std.err
(Intercept)    0.516  0.0944
Number of clusters:   102  Maximum cluster size: 1

[[7]]

Call:
geeglm(formula = IL_23 ~ Time + Supplemented_dose + Age_years,
    data = ., id = Lp)

 Coefficients:
                  Estimate Std.err  Wald Pr(>|W|)
(Intercept)         -0.3901  0.9244  0.18     0.67
Time               3.6882  0.4217 76.49   <2e-16 ***
Supplemented_dose    -0.5156  0.4449  1.34     0.25
```

```
Age_years           0.0231  0.0188  1.52     0.22
---
Signif. codes:  0 ‘***’ 0.001 ‘**’ 0.01 ‘*’ 0.05 ‘.’ 0.1 ‘ ’ 1

Correlation structure = independence
Estimated Scale Parameters:

            Estimate Std.err
(Intercept)     4.54   0.727
Number of clusters:   102  Maximum cluster size: 1

[[8]]

Call:
geeglm(formula = sCD40L ~ Time + Suplementowal_dawke + Wiek_lata,
    data = ., id = Lp)

 Coefficients:
                  Estimate Std.err Wald Pr(>|W|)
(Intercept)          692.05  306.98 5.08    0.024 *
Time               -270.21  143.04 3.57    0.059 .
Supplemented_dose    -264.58  175.23 2.28    0.131
Age_years             8.15    8.42 0.94    0.333
---
Signif. codes:  0 ‘***’ 0.001 ‘**’ 0.01 ‘*’ 0.05 ‘.’ 0.1 ‘ ’ 1

Correlation structure = independence
Estimated Scale Parameters:

            Estimate Std.err
(Intercept)   521725  152955
```

Number of clusters: 102 Maximum cluster size: 1

[[9]]

Call:

```
geeglm(formula = TNF_α ~ Time + Supplemented_dose + Age_years,
    data = ., id = Lp)
```

Coefficients:

	Estimate	Std.err	Wald	Pr(>|W|)
(Intercept)	-0.134	0.764	0.03	0.86
Time	0.240	0.356	0.46	0.50
Supplemented_dose	-0.483	0.420	1.33	0.25
Age_years	0.026	0.018	2.07	0.15

Correlation structure = independence

Estimated Scale Parameters:

	Estimate	Std.err
(Intercept)	3.23	0.991

Number of clusters: 102 Maximum cluster size: 1

Stratified by Sex_0_woman__1__man

	0	1	p	test
n	66	38		
25OHD (median [IQR])	29.65 [24.06, 34.40]	24.94 [17.84, 32.25]	0.075	nonnorm
IL_4 (median [IQR])	5.92 [3.78, 8.26]	7.07 [3.78, 9.29]	0.659	nonnorm
IL_10 (median [IQR])	2.36 [0.96, 4.08]	2.23 [0.96, 4.04]	0.709	nonnorm

IL_25 (median [IQR])	0.16 [0.04, 0.34]	0.30 [0.04, 0.41]	0.098	nonnorm
IL_1β (median [IQR])	2.08 [0.53, 3.90]	2.08 [0.57, 5.33]	0.278	nonnorm
IL_6 (median [IQR])	0.82 [0.46, 1.13]	0.79 [0.46, 1.13]	0.678	nonnorm
IL_17A (median [IQR])	0.23 [0.21, 0.27]	0.23 [0.21, 0.27]	0.702	nonnorm
IL_17F (median [IQR])	16.92 [0.55, 20.83]	19.52 [0.58, 23.43]	0.197	nonnorm
IL_21 (median [IQR])	11.46 [2.71, 21.22]	17.69 [2.71, 22.40]	0.976	nonnorm
IL_22 (median [IQR])	4.87 [2.65, 7.58]	4.65 [2.65, 7.58]	0.976	nonnorm
IL_23 (median [IQR])	5.11 [4.06, 7.51]	5.04 [3.66, 7.91]	0.733	nonnorm
IFN_γ (median [IQR])	1.22 [0.19, 2.11]	1.36 [0.01, 2.25]	0.886	nonnorm
TNF_α (median [IQR])	0.50 [0.11, 0.62]	0.50 [0.12, 1.68]	0.488	nonnorm
IL_31 (median [IQR])	260.32 [106.49, 553.96]	392.76 [128.95, 868.08]	0.105	nonnorm
IL_33 (median [IQR])	4.73 [0.54, 17.80]	4.71 [0.56, 18.18]	0.997	nonnorm
sCD40L (median [IQR])	258.98 [13.27, 555.00]	324.28 [10.70, 774.42]	0.671	nonnorm
Stosunek_IL_17A_IL_10 (median [IQR])	0.16 [0.05, 0.28]	0.22 [0.05, 0.28]	0.648	nonnorm
Stosunek_IL_6_IL_10 (median [IQR])	0.52 [0.12, 1.22]	0.58 [0.12, 1.16]	0.559	nonnorm
CER (median [IQR])	40.62 [36.61, 48.50]	36.82 [33.59, 43.16]	0.015	nonnorm
SH (median [IQR])	260.74 [232.44, 306.74]	263.42 [235.50, 298.11]	0.925	nonnorm
SH_bialko (median [IQR])	3.50 [3.25, 3.82]	3.47 [3.12, 3.99]	0.941	nonnorm
TAC (median [IQR])	1.02 [0.93, 1.12]	1.06 [0.98, 1.14]	0.184	nonnorm

TOS (median [IQR])	17.29 [13.82, 21.23]	15.11 [12.24, 23.95]	0.710	nonnorm
LPH (median [IQR])	5.39 [3.87, 7.03]	5.78 [3.80, 8.13]	0.432	nonnorm
SOD (median [IQR])	19.45 [18.39, 20.53]	19.57 [18.36, 20.48]	0.925	nonnorm
MnSOD (median [IQR])	11.63 [10.72, 12.62]	11.28 [10.67, 11.88]	0.283	nonnorm
CuZnSOD (median [IQR])	8.17 [7.01, 9.23]	8.18 [7.03, 9.37]	0.438	nonnorm
LPS (median [IQR])	102.39 [85.39, 121.68]	108.71 [91.81, 118.73]	0.566	nonnorm
MDA (median [IQR])	1.70 [1.43, 2.06]	1.84 [1.49, 2.23]	0.240	nonnorm
ALCAM (median [IQR])	2.03 [1.43, 3.07]	2.21 [1.48, 3.19]	0.700	nonnorm
CXCL16 (median [IQR])	0.26 [0.00, 0.56]	0.28 [0.00, 0.66]	0.790	nonnorm
PTX_3 (median [IQR])	2367.83 [1842.05, 2940.82]	2180.01 [1713.28, 2712.18]	0.207	nonnorm
IL_1RA (median [IQR])	1075.76 [762.11, 1853.82]	1207.80 [726.23, 1789.04]	0.808	nonnorm
OPG (median [IQR])	6.07 [5.01, 7.10]	6.02 [5.01, 6.94]	0.914	nonnorm
